# Synthesis and Characterization of Biocompatible Methacrylated Kefiran Hydrogels: Towards Tissue Engineering Applications

**DOI:** 10.3390/polym13081342

**Published:** 2021-04-20

**Authors:** Hajer Radhouani, Susana Correia, Cristiana Gonçalves, Rui L. Reis, Joaquim M. Oliveira

**Affiliations:** 1 3B’s Research Group, I3Bs—Research Institute on Biomaterials, Biodegradables and Biomimetics, University of Minho, Headquarters of the European Institute of Excellence on Tissue Engineering and Regenerative Medicine, AvePark, Parque de Ciência e Tecnologia, Zona Industrial da Gandra, Barco, 4805-017 Guimarães, Portugal; susana.correia@i3bs.uminho.pt (S.C.); cristiana.goncalves@i3bs.uminho.pt (C.G.); rgreis@i3bs.uminho.pt (R.L.R.); miguel.oliveira@i3bs.uminho.pt (J.M.O.); 2 ICVS/3B’s–PT Government Associate Laboratory, Braga, 4805-017 Guimarães, Portugal

**Keywords:** biomedical applications, chemical cross-linking, hydrogel, Kefiran, methacrylation

## Abstract

Hydrogel application feasibility is still limited mainly due to their low mechanical strength and fragile nature. Therefore, several physical and chemical cross-linking modifications are being used to improve their properties. In this research, methacrylated Kefiran was synthesized by reacting Kefiran with methacrylic anhydride (MA). The developed MA-Kefiran was physicochemically characterized, and its biological properties evaluated by different techniques. Chemical modification of MA-Kefiran was confirmed by ^1^H-NMR and FTIR and GPC-SEC showed an average Mw of 793 kDa (PDI 1.3). The mechanical data obtained revealed MA-Kefiran to be a pseudoplastic fluid with an extrusion force of 11.21 ± 2.87 N. Moreover, MA-Kefiran 3D cryogels were successfully developed and fully characterized. Through micro-CT and SEM, the scaffolds revealed high porosity (85.53 ± 0.15%) and pore size (33.67 ± 3.13 μm), thick pore walls (11.92 ± 0.44 μm) and a homogeneous structure. Finally, MA-Kefiran revealed to be biocompatible by presenting no hemolytic activity and an improved cellular function of L929 cells observed through the AlamarBlue^®^ assay. By incorporating methacrylate groups in the Kefiran polysaccharide chain, a MA-Kefiran product was developed with remarkable physical, mechanical, and biological properties, resulting in a promising hydrogel to be used in tissue engineering and regenerative medicine applications.

## 1. Introduction

Achieving translational medicine together with commercial success requires regenerative polymers to be not only safe and appropriate but also cost-effective and suitable for production and biomedical/clinical use. Tissue engineering and regenerative medicine (TERM) strategies have shown to be promising to regenerate damaged tissues and to help regain normal body functions [1]. Biodegradable natural polymers are currently widely used for engineered tissue devices with their unique structural and compositional similarities with the natural extracellular matrix (ECM). A key advantage of using these polymers is their capability to successfully promote cell growth in vitro and regenerate tissue in vivo, without toxicity or immunological reactions, which make them attractive for TERM strategies [2,3]. Hydrogels have received significant attention in the TERM field, principally due to their ability to be 3D biological materials that mimic aspects of a certain ECM so that the tissue’s functions can be improved, maintained, or restored [4]. A main advantage of hydrogels is their ability to fully recreate in situ the complexity of a tissue defect. Being polymeric networks with high-water content, 3D hydrogels have played a pivotal microenvironment for cell colonization with their mechanical and biochemical properties [5]. In the last decade, engineered 3D scaffolds have been successfully produced through several techniques such as phase separation [6], lithography [7], freeze-drying [8], cryogelation [9], salt leaching [10], gas foaming (supercritical CO_2_ drying) [11,12], among others. It is important to highlight that when using water as solvent for scaffold fabrication, a special care is needed regarding pore collapse upon its removal. As such, either supercritical [13] or freeze–drying [8] is normally used to guarantee pore stabilization within the final porous biomaterials [12].

Though, the feasibility of applying hydrogels is still limited including the fact that they have low mechanical strength and a fragile nature. Therefore, new approaches are being investigated to improve the in vivo application of hydrogels, including physical and/or chemical cross-linking to enhance these properties, as well as functionalization to better mimic the native extracellular environment [14].

Physical and chemical cross-linking mechanisms have been achieved for several natural-origin hydrogels, such as agar, alginate, gelatin, gellan gum, and hyaluronic acid [4]. Regarding the physical synthesizing method, hydrogen bond formation, hydrophobic interaction, and ionic interaction were the most used; on the other side, the chemical cross-linking approach that relies on covalent bond formation includes different polymerization methods such as step-growth polymerization, irradiation polymerization, and chain-growth polymerization [15]. A suitable hydrogel for biomedical applications demands specific important criteria that will define the final properties of the device such as elasticity and mechanical strength. Furthermore, the new material needs to be biocompatible and non-cytotoxic to the corresponding tissue. However, these biopolymer-based hydrogels can sometimes present a certain weakness and, more importantly, lack the adequate mechanical properties that can be modified through either physically or chemically in situ cross-linking reactions. Microbial polysaccharides, a renewable resource, have received great interest in the TERM areas, particularly in producing new biomaterials. Recently, Kefiran-based scaffolds were produced through a freeze gelation technique [16]. In previous research, Kefiran, an exopolysaccharide produced by lactic acid bacteria from kefir grains, has received particular attention for biomedical uses [16,17,18].

In the present work, Kefiran was chemically modified by reacting this polysaccharide with methacrylic anhydride (MA) to be used in tissue engineering applications. Thus, the obtained MA-Kefiran hydrogel was fully characterized for its structural, physicochemical, and biological properties through a complete set of technologies such as ^1^H-NMR (^1^H nuclear magnetic resonance spectroscopy), FTIR (Fourier-transform infrared spectroscopy), DSC (differential scanning calorimetry), GPC-SEC (gel permeation chromatography − size exclusion chromatography), SEM (scanning electron microscopy), micro-CT (micro-computed tomography), and rheology. The cytocompatibility of the developed MA-Kefiran hydrogel was also evaluated by quantifying its hemolytic activity and its impact on the metabolic activity of exposed L929 cells.

## 2. Materials and Methods

### 2.1. Production of Kefiran Polysaccharides

Kefiran exopolysaccharides were isolated from kefir grains as previously described by our group [19]. These kefir grains were purchased locally from a household in Guimarães, Portugal. Twenty grams of kefir grains were treated in water at 60 °C (1:10) for 30 min, and the mixture was centrifuged at 18,300× *g* (20 min, 20 °C). From the supernatant, the polysaccharide was precipitated by two volumes of cold ethanol and left at −20 °C overnight, and a second centrifugation at 18,300× *g* (20 min, 4 °C) was performed. Pellets were completely dissolved in water and this precipitation procedure was repeated twice. Finally, the precipitates were re-dissolved in water (60 °C) and the obtained concentrated solution was the crude polysaccharide. The polysaccharide samples were finally freeze-dried.

### 2.2. Synthesis of Methacrylated Kefiran

Methacrylated Kefiran (MA-Kefiran) was synthesized by reacting the Kefiran polysaccharide with methacrylic anhydride (MA) (Sigma-Aldrich Química S. L., Sintra, Portugal) [19].The Kefiran polysaccharide (0.2 g) was dissolved into 20 mL of PBS (5 mM, pH 7.4) and to this solution, 0.392 mL of MA was added. Then, the pH 8.5 of the solution was continuously readjusted with a NaOH solution (1 M), and the reaction was continued under constant stirring for 2.5 h. Finally, the MA-Kefiran solution was precipitated with 3 volumes of cold acetone and purified by dialysis for 7 days to remove the excess of MA and acetone. The obtained MA-Kefiran samples were lyophilized.

### 2.3. Preparation of MA-Kefiran Scaffold

To produce MA-Kefiran cryogels, a MA-Kefiran polysaccharide solution was prepared at a concentration of 2% (*w/V)* in ultrapure H_2_O. A total of 200 µL of the solution was transferred to 96-well plate molds and placed immediately at −20 °C for 24 h and at 4 °C for a further 24 h. Finally, the obtained Kefiran cryogels were freeze-dried.

### 2.4. Physicochemical Characterization of MA-Kefiran

#### 2.4.1. H nuclear Magnetic Resonance Spectroscopy (NMR)

The chemical modification of Kefiran was assessed by ^1^H-NMR (Bruker, Massachusetts, USA). The methacrylation degree (DM) which corresponds to the fraction of modified hydroxyl groups per repeating unit) was calculated through the following equation:

(1) (Imethylene ICH3nCH3×nmethylene )nOH

$I_{\mathrm{methylene}}$—Relative integration of the methylene proton peak of methacrylate groups;

$I_{{\mathrm{CH}}_{3}}$—Relative integration of the methyl protons of the initial standard;

$n_{\mathrm{methylene}}$—Number of protons in the CH_2_ = from the methacrylic group;

$n_{{\mathrm{CH}}_{3}}$—Number of protons in the CH_2_ = in the methyl groups of the Kefiran structure;

$n_{\mathrm{OH}}$—Number of reactive -OH sites per sugar residue in the Kefiran structure.

#### 2.4.2. Fourier-Transform Infrared Spectroscopy (FTIR)

The transmission spectra of MA-Kefiran were acquired on an IR Prestige-21 spectrometer (Shimadzu, Kyoto, Japan), using 32 scans, with a wavenumber range between 4000 and 600 cm^−1^ and a resolution of 4 cm^−1^.

#### 2.4.3. Gel Permeation Chromatography—Size Exclusion Chromatography (GPC-SEC)

The molecular weight of MA-Kefiran was determined by GPC-SEC (Malvern Panalytical, Malvern, UK). The measurements were performed at 30 °C with a Malvern ViscoteK TDA 305 by means of using refractometer, right and low angle light scattering and viscometer detectors on a set of four columns: pre-column Suprema, 5 µm, 8 × 50, Suprema 30 Å, 5 µm, 8 × 300, and 2× Suprema 1000 Å, 5 µm 8 × 300. We have used PBS buffer saline (0.01 M PBS, 0.0027 M KCl and 0.137 M NaCl, pH 7.4, at 25 °C) and 0.05% (*w/V*) NaN_3_ at a rate of 1 mL/min. The absolute molecular weight was evaluated by a calibration of the LS and RI detectors obtained using the Omnisec 5.12 software (ViskoteK) using pullulan (Mn, 48.8 kDa and PDI 1.07). The dn/dc of the polysaccharide was set to 0.15.

#### 2.4.4. Differential Scanning Calorimetry (DSC)

The thermal analysis was carried out using a TA-Q100 equipment (Jasco, Tokyo, Japan). MA-Kefiran samples (5–10 mg) were prepared and filled into aluminum pans. For DSC reference, an empty aluminum pan was used and run simultaneously. Under a nitrogen atmosphere, the samples were heated in two stages from 20 °C to 250 °C with a continuous rate of 20 °C /min; then were left during 2 min at this temperature, and then, they were cooled at 20 °C /min to the initial temperature; a second heating run was conducted.

#### 2.4.5. Rheology

A Kinexus pro + rheometer (Malvern Instruments, Malvern, UK) equipped with a 316-grade stainless steel cone-plate system composed by a plate lower pedestal and an upper measurement geometry cone with 40 mm of diameter and 4° angle was used for rheological analyses using the acquisition software rSpace. The following experiments were conducted with MA-Kefiran samples at 1% (*w/V*) in H_2_O. The plots were obtained by means of applying an average of experiments (*n* = 3).

The rotational experiment determines the shear viscosity as a function of the shear rate, from 0.01 s^−1^ to 1000 s^−1^ at 37 °C.

The oscillatory experiment evaluates the viscoelasticity behavior of the material, through frequency sweep curves.

The pull away experiment evaluates the adhesion properties of MA-Kefiran, by calculating the maximum force required to pull the plate away from the sample. The conditions of the experiment were a gap speed of 1 mm/s, a contact force of 1 N and a contact time of 2 s.

#### 2.4.6. Injectability Assay

The injectability test of the MA-Kefiran sample was carried out by an injection measurement device. This equipment (KD Scientific, Leiria, Portugal) was composed of a syringe pump where was placed a 1 mL disposable syringe with a needle gauge of 21, and the assay was realized in extrusion mode with a rate of 1 mL/min.

#### 2.4.7. Micro-Computed Tomography (Micro-CT) Analysis

The microstructure of the prepared MA-Kefiran scaffold (*h* 8 mm × *d* 5 mm) was evaluated using a high-resolution X-ray microtomography system Skyscan 1272 scanner (Bruker Micro-CT, Kontich, Belgium). Each MA-Kefiran sample was scanned using a pixel size of 5 μm, a rotation site of 0.45° over a rotation range of 360°. Images were acquired with no filter, 50 kV X-ray source of energy, 200 μA current and a resolution of 2452 × 1640 pixels. A dynamic threshold of 20 was used to convert the reconstructed grey-scale images into binary images that were then used for morphometric analysis by quantifying porosity, interconnectivity, mean pore size and mean wall thickness (CT Analyzer v1.12.0.0, SkyScan, Kontich, Belgium).The scaffold cross-sectional and longitudinal cuts and 3D virtual models were also created, visualized and registered using the image processing and reconstruction software’s Data Viewer (v1.6.6.0) and CT-Vox (v2.0.0) (SkyScan, Kontich, Belgium), respectively.

#### 2.4.8. Scanning Electron Microscopy (SEM)

MA-Kefiran scaffolds were fixed with carbon tape to aluminum stubs and platinum-coated in a sputter coater (EM ACE600, Leica, Wentzler, Germany). Morphology images were obtained on a JSM-6010LV SEM (JEOL, Tokyo, Japan), featuring integrated energy dispersive spectroscopy (EDS) (INCAx-Act, PentaFET Precision, Oxford Instruments, Abingdon-on-Thames, UK).

### 2.5. Biological Characterization of MA-Kefiran

#### 2.5.1. Hemolytic Assay

Five mL of rabbit blood (previously extracted with citrate dextrose) were added to 45 mL of PBS. During 10 min, the samples were harvested by centrifugation (1000× *g*, 4 °C) and the supernatant was then discarded. This process was repeated two more times until the PBS solution was clear, thus indicating that the pellet was clean. PBS was then added to make a solution of 3% (*w/V*). Then, in each well of a 96-well plate, 80 µL of rabbit blood was deposited along with 80 µL of each sample. Three wells, each containing blood with PBS and Triton X-100 were used as negative and positive controls, respectively. During 4 h the plates were incubated at 37 °C, and afterwards centrifuged at 1000× *g* for 10 min, at 4 °C. Finally, the supernatant (80 µL) of each well was transferred into a 96-well-U-bottom cell culture plate and the absorbance at 558 nm (ABS) was measured. For each sample, data was normalized with the controls, using the following formula:

Hemolysis (%) = [ (ABS_sample_/ABS_triton_) − 1] × 100 (2)

This research has been reviewed by the Animal Welfare Body of the School of Medicine and its Life and Health Science Research Institute (EM/ICVS), and the Research Institute on Biomaterials, Biodegradables and Biomimetics (I3Bs) - ORBEA EM/ICVS-I3Bs (Reference: ORBEA EM/ICVS-I3Bs_002/2019) as well as by the National Competent Authority Direção-Geral de Alimentação e Veterinária (DGAV) (Reference: 0421/000/000/2020 - 022434). The research has been approved by ORBEA on November 13th of 2019 and by DGAV on December 14th of 2020.

#### 2.5.2. Cytotoxicity Assessment

The biocompatibility of MA-Kefiran was assessed using the L929 cell line, as described in ISO 10993-5 (2009). In brief, 10,000 cells per well were seeded in 96-well plates and a 4% (*w/V*) MA-Kefiran solution was prepared in 0.9% (*w/V*) of NaCl, as specified in ISO 10993-12 (2012). The culture medium was composed of low-glucose Dulbecco’s Modified Eagle Medium (DMEM, Sigma-Aldrich, Saint Louis, MO, USA) supplemented with 10% (*v/V*) fetal bovine serum (FBS, Invitrogen, Carlsbad, CA, USA) and 1% (*v/V*) antibiotic/antimycotic (Invitrogen). After 24 h, the culture medium of each well was replaced for the Kefiran solution diluted in culture medium at a final concentration of 1% (*v/V*). A sample containing only culture medium was used as negative control and a 1% (*v/V*) Triton X-100 (Sigma-Aldrich, St. Louis, MO, USA) solution prepared in culture medium was used as a positive control. Cultures were maintained at 37 °C under a humidified atmosphere of 5% (*v/V*) CO_2_ in air. Finally, at 24 h, 48 h and 72 h of culture, cell proliferation was assessed through the AlamarBlue^®^ assay (Bio-Rad, Hercules, CA, USA) and total double-stranded DNA (dsDNA) quantification.

Cell metabolic activity of L929 was assessed using the AlamarBlue^®^ assay (Bio-Rad). AlamarBlue^®^ has resazurin in its composition, which is reduced to resorufin that upon entering living cells results in a highly fluorescent compound that can be easily detected. At each time point, the cell cultures were incubated with AlamarBlue^®^ reagent (10% *v/V*) in DMEM for 3 h at 37 °C. Cell suspensions were then distributed into wells of a 96-well black plate, and fluorescence measurements were made using a Biotek Synergy HT microplate reader with Ex/Em at 530/590 nm. To quantify the total dsDNA, at each time point, the samples were collected and incubated for 1 h at 37 °C in ultrapure H_2_O, and then stored at −80 °C until analyzed. Using the Quant-iT PicoGreen dsDNA kit (Molecular Probes, Invitrogen) quantification assay, the dsDNA samples were directly transferred to a 96-well white plate where they were diluted and mixed with TE buffer. After the addition of Quant-iT PicoGreen dsDNA reagent, samples were incubated at room temperature in the dark and then fluorescence was measured with Ex/Em at 480/530 nm. A standard DNA curve in the range of 1–2000 ng/mL was used to convert RFUs into ng/mL.

### 2.6. Statistical Analysis

Data analysis was performed utilizing the software Origin 2018 (Originlab Corporation, Northampton, MA, USA) for Windows. Shapiro–Wilk test (significance level of 0.05) was used for the assessment of the normality of data. Comparisons between groups with normal distribution were made using two-way ANOVA supplemented by Tukey’s test. All data are expressed as mean ± standard deviation.

## 3. Results

### 3.1. H-NMR

**Figure 1:**
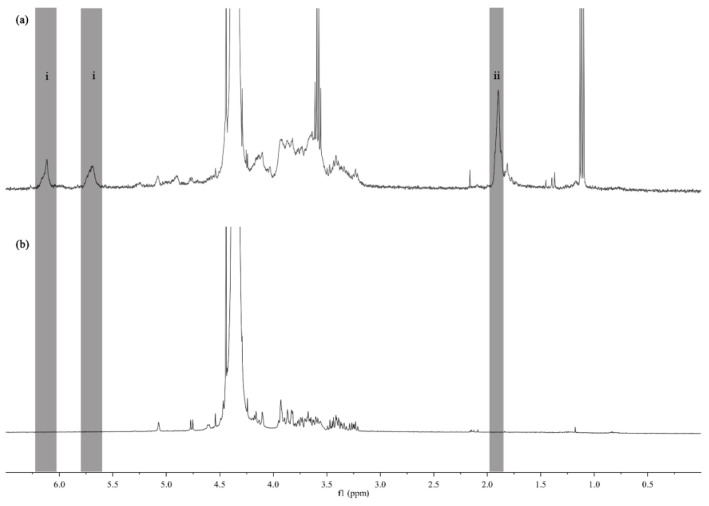
^1^H nuclear magnetic resonance (^1^H-NMR) spectrum of methacrylated Kefiran (**a**) and Kefiran (**b**) in deuterium oxide (D_2_O) at 60 °C. The vinyl groups of the methacrylic anhydride (MA) were identified around δ 5.6–6.4 ppm (i) and the methyl group of MA was located around δ 1.9 ppm (ii).

Quantitative NMR has become a widely used analytical tool for the quantification of polysaccharides, being an important tool when evaluating the chemistry of these polymers [20]. In our study, the chemical modification of Kefiran was assessed by ^1^H-NMR (Figure 1a). The ^1^H-NMR spectrum revealed several anomeric α hydrogen, assigned to a sugar on a lateral branch (Figure 1b), at the chemical shifts around 4.62 ppm, 4.67 ppm, 4.76 ppm, 4.78 ppm, 4.83 ppm, and 4.85 ppm. Furthermore, an anomeric *β* hydrogen through a peak at 5.15 ppm was also observed. These data were also confirmed in a previous research [19].

The methacrylation of Kefiran was confirmed through the ^1^H-NMR spectrum and the degree of substitution obtained for the synthesized material MA-Kefiran was 47.6 ± 3.8%. A previous study showed the methacrylation of the gellan gum (MA-GG) polysaccharide [4] but the degree of substitution was considerably lower than that obtained in MA-Kefiran.

### 3.2. FTIR

**Figure 2:**
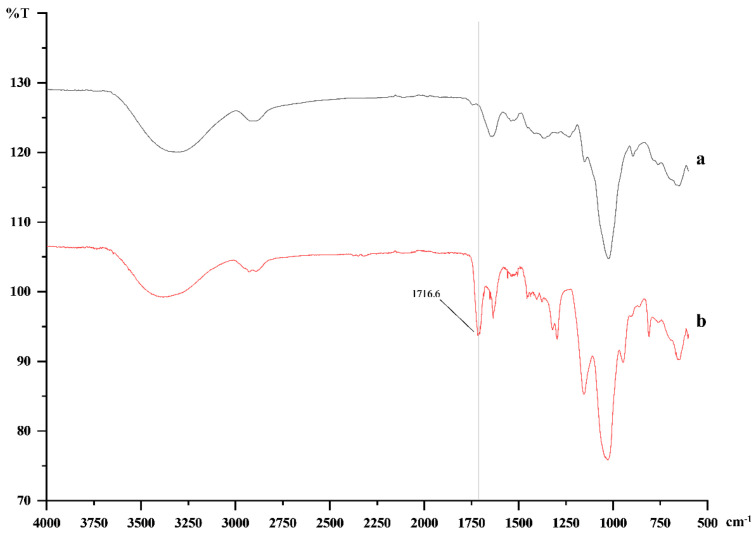
Fourier-transform infrared spectroscopy (FTIR) spectra of Kefiran (**a**) and methacrylated-Kefiran (**b**). The shoulder appearing at 1717 cm^−1^ reveals the presence of the carbon double bond peak of methacrylate groups in the modified Kefiran.

FTIR has been used to identify the fundamental groups present in polymer structures; it represents a useful tool to study structural changes in polysaccharides, which is important for the evaluation of their biodegradability, biocompatibility, and biological response [21]. The FTIR spectrum has also proven the methacrylation of Kefiran by the appearance of the carbon double bond peak at 1717 cm^−1^, identified in methacrylate groups but not in the unmodified Kefiran polysaccharide (Figure 2).

The use of polysaccharides for TERM is dependent on their biological and physical properties; also, the presence of reactive chemical functional groups make polysaccharides easily flexible to physical and chemical cross-linking, which turn them ideal biomaterials for tissue engineering applications [22]. As previously demonstrated, the molecular structure and component dynamics of materials were studied by NMR and FTIR.

### 3.3. DSC

**Figure 3:**
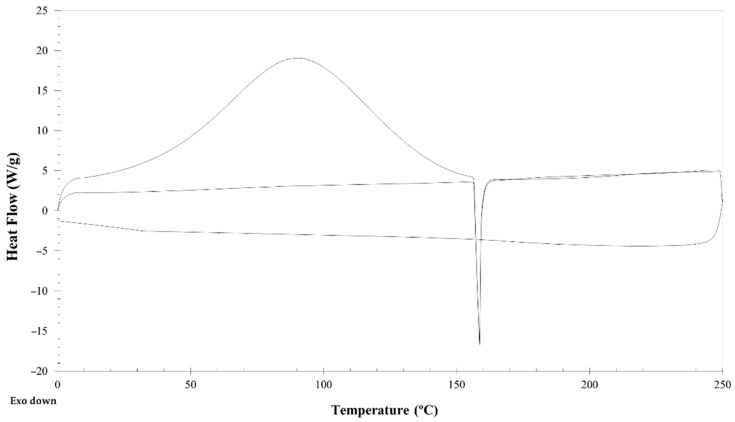
Differential scanning calorimetry (DSC) spectra of methacrylated-Kefiran showing a sharp endothermic peak at 92 °C corresponding to the water evaporation of the sample, and an exothermic peak at 159 °C that could be related to the rapid decomposition behavior of MA-Kefiran.

DSC is the most employed thermal analysis tool to evaluate the thermophysical and kinetic properties of materials with temperature. DSC is used to assess the heat fusion, melting temperature, glass transition temperature, reaction energy and temperature, denaturation temperature, among others [23]. Thus, MA-Kefiran was characterized to assess its thermal stability (Figure 3).

Figure 3 presented the MA-Kefiran thermogram (endothermic heat flow); the functionalized MA-Kefiran showed lower thermal stability than the native Kefiran polysaccharide that previously reported a degradation temperature of 273 °C [19]. The observed difference between the native and the modified Kefiran regarding the thermal property is not significant since the developed biomaterial will be used at physiological temperatures.

Recent studies, which developed an injectable, also used thermal analysis tools to assess the molecular structure and purity of Kefiran polysaccharides extracted from commercial and traditional kefir products. These hydrogels based on Kefiran were aimed to be used as potential scaffolds for tissue regeneration or implantable drug delivery devices [17,24].

### 3.4. GPC-SEC

This functionalized MA-Kefiran polymer presented a number-average molecular weight and a weight-average molecular weight of 793 kDa and 620 kDa, respectively, and a polydispersity index (PDI) of 1.3. It is important to highlight that the PDI, indicating the distribution of polymer chain molecular weights, is usually in the range of 1.2 to 1.5 for polysaccharides from natural resources.

The data obtained through GPC-SEC are considered significant parameters for the biomaterial application. In fact, these data will define the physicochemical properties of polymers such as solubility, viscosity, degradation, mechanical properties, among others, and the particular application of a certain polymer will be related to these properties. For example, a polymer with low molecular weight will have short degradation rate; and the degradation rate of a biomaterial is commonly matched with the rate of tissue regeneration. Moreover, a material with a high molecular weight will have a better thermal stability even at very high temperatures, and will increase its mechanical properties [25,26,27].

### 3.5. Rheology of MA-Kefiran Extract

**Figure 4:**
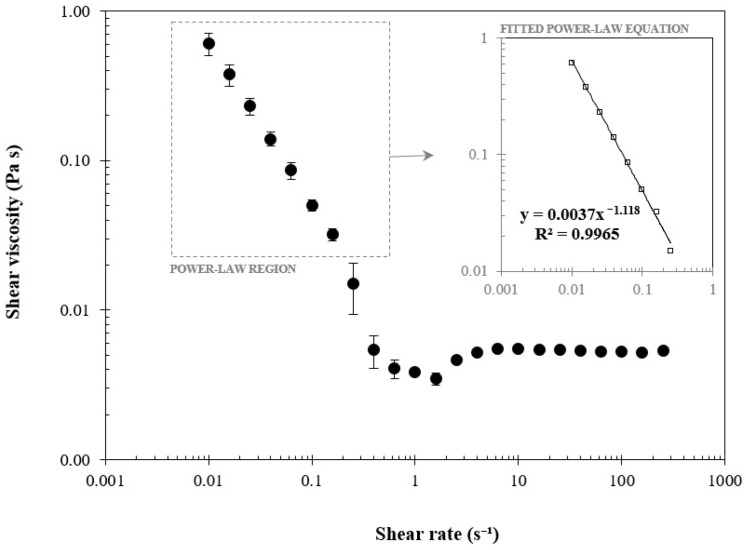
Shear viscosity versus shear rate of methacrylated-Kefiran at 1% (*w/V*). The indexes of consistency (*K*, 0.003) and flow behavior (*n*, 1.118) in the power-law equation indicate a pseudoplastic behavior.

To perform the mechanical tests for the MA-Kefiran extract under simulated physiological conditions the samples were measured immediately under 37 °C. The flow behavior of a 1% (*w/V*) MA-Kefiran solution is illustrated in Figure 4. This plot shows data of the shear viscosity measured as function of shear rate.

It has been shown that the viscosity declined as the shear rate augmented in MA-Kefiran at 37 °C, which corresponds to a shear thinning fluid. This characteristic was also observed previously in Kefiran solutions (1% and 10% *w/V*) at 37 °C [17].

This trend could be explained by the fact that polymers with large molecular chains tumble randomly which will affect the polymer’s fluidity under low shear. Thus, the flow-aligning effect of these biomolecules with the increase of shear will present less resistance to the shear [28].

From the perspective of an injectable biomaterial for tissue engineering application, the information obtained from the viscosity upon shear rate is important as it is highly desirable that the viscosity of these biomaterials quickly decreases when exposed to the increase of the shear rate in order to have low resistance during the injection process [29]. Mechanical properties are evaluated in terms of storage and loss moduli, and gel strength. In fact, as showed in this research, these properties can be modulated by changing the molecular weight of the polymer through incorporation of additional components.

### 3.6. Injectability Assay

Injectability is considered a key-product performance indicator for parenteral dosage forms. This measurement refers to the performance of the formulation during injection. In fact, this concept is an important parameter for specialized dosage forms, like suspensions, injectable emulsions, microemulsions, liposomes, and microspheres [30]. In this work, MA-Kefiran showed an extrusion force of 11.21 ± 2.87 N, a value similar to hyaluronic acid (11.3 N) [17]. The latter polymer is gaining popularity as a viscosupplementation product in the management of osteoarthritis (OA) [31]. Intra-articular injections have emerged as a promising strategy of non-surgical treatment of an appreciable number of OA patients, especially in those with mild-to-moderate OA [32].

### 3.7. Production of MA-Kefiran Scaffolds

Tissue engineering also contributes to the regeneration and repair of injured tissues by joining autologous cells with an adequate scaffold biomaterial. This scaffold acts as natural template for TE in guiding the growth and the remodeling of new functional tissue [33]. Usually, the structure of the polymeric scaffold is not easy to evaluate in terms of pore shape and size, particularly in three dimensions [34].

### 3.8. Assessment of the MA-Kefiran Scaffolds’ Morphological Properties

In this research, to explore, visualize, and analyze in detail the 3D structure of the MA-Kefiran scaffold, micro-CT and SEM were used as powerful platforms [35].

#### 3.8.1. Micro-CT

**Figure 5:**
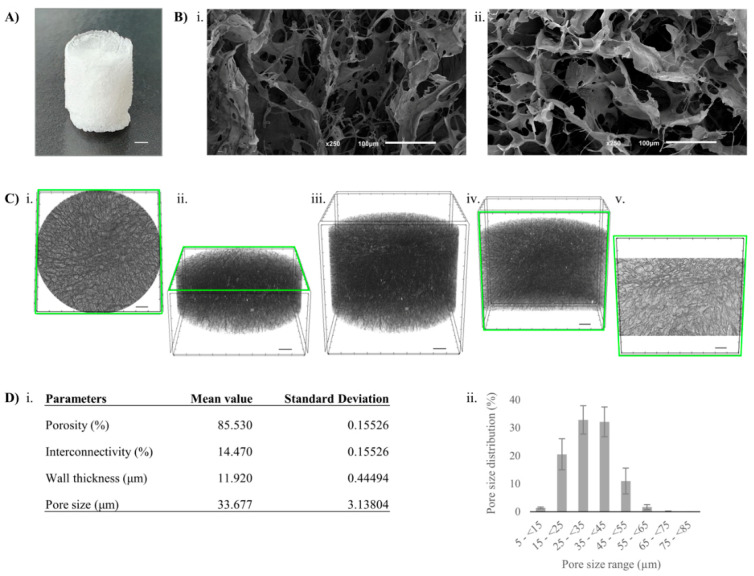
Morphology of methacrylated-kefiran scaffolds after freeze-drying. (**A**) Macroscopic image (scale bar: 1 mm); (**B**) scanning electron microscopy (SEM) microstructure of surface at ×250 of magnification: longitudinal section (i); cross section (ii); (**C**) 3D reconstructed MA-Kefiran scaffolds using the micro-computed tomography (CT) system: cross-sectional (i, ii), longitudinal (iv, v) and fully reconstructed (iii) views (scale bars: 500 µm); (**D**) micro-CT data: structure characterization (i) and pore size distribution (ii).

The key parameters to consider when optimizing a scaffold is to create a biomaterial with balanced biological and physical properties with certain structural parameters regarding porosity, pore morphology, pore size and pore distribution [36]. In this work, the pore diameter and distribution of the developed MA-Kefiran scaffold were evaluated by micro-CT. The data obtained revealed a high porosity for this scaffold (85.53 ± 0.15%) (Figure 5).

From the quantitative analysis of micro-CT, it was possible to determine the porosity, wall thickness and pore size of the MA-Kefiran scaffold (85.5%, 11.9 μm and 33.7 μm, respectively). Moreover, the high porosity and the high surface area to volume ratio are required for homogeneous cell delivery, cellular attachment, and newly-formed tissue ingrowth [37].

Micropores (33 μm) such those observed in our research, are characterized by a larger specific surface area, making cell attachment and protein adhesion on the 3D scaffold more favorable, while larger and interconnected pores (macropores) improve cellularity and ECM ingrowth for faster constructs development [38]. In soft tissue applications, pore sizes of 35 μm demonstrated success in promoting vascularization when compared to 20 μm and 70 μm pores [33]. Moreover, a previous research revealed that the ideal pore size for initial cell adhesion was 95 mm in vitro [39]. Though, in bone tissue engineering, the pore range of 300–400 μm has been revealed to be more appropriate for osteoblast attachment, proliferation, and differentiation [40]. It is noteworthy that, for cell infiltration and host tissue ingrowth and vascularization, there is an optimal pore size range, such as 5 µm for neovascularization [41], 5–15 µm for fibroblasts [42], 20–125 µm for adult mammalian skin tissues [43], 40–100 µm for osteoid tissues [44] and 100–400 µm for bone tissues [45], for a suitable implanted biomaterial.

The morphology and porosity characteristics such as their size, volume, interconnectivity, shape, among others, must be cautiously tailored during scaffold production. These properties are essential for new tissue regeneration, including building the cellular network and interconnected pathways not only for oxygen, nutrient and regulatory factor transportation, but also for cell signaling, differentiation and proliferation [46].

It is important to highlight that the equilibrium must be well-known between obtaining optimal cell attachment, proliferation and differentiation, thus facilitating the new tissue growth [47].

#### 3.8.2. SEM

SEM analysis, a precise tool, is considered one of the most widely used in TE [48]. SEM analysis of the MA-Kefiran scaffold showed a foam-like structure and a smooth surface uniformly distributed. Figure 5 demonstrates the change in porosity and structure in the chemically modified Kefiran polymer. Visually, the freeze-dried modified scaffold has a more compact structure, which was verified by the data observed in SEM and micro-CT. Moreover, it is important to reinforce that the pore after the freeze-drying process reveals a very homogeneous structure and shows the ice-crystal morphology [49]. It is well known that the architectural parameters of a suitable scaffold can be achieved by various techniques that will provide a different level of control of pore size, distribution, and connectivity; for instance, supercritical drying commonly leads to smaller pores (usually meso and micropores) and in the other side, freeze–drying, which in turn leads to macroporous structures [50]. Thus, it will be interesting to perform in the future different processing methodologies, perhaps more advanced ones such as 3D printing or supercritical fluid technologies, in order to optimize the scaffold’s performance for a certain TERM application [13,51].

The morphology of the TE scaffold is a key aspect that affects the migration, proliferation, and differentiation of cells. The ideal scaffold for TE is a biomaterial platform with well-adjusted architectural, biological, mechanical and physical properties [52].

### 3.9. Rheology of MA-Kefiran Cryogel

**Figure 6:**
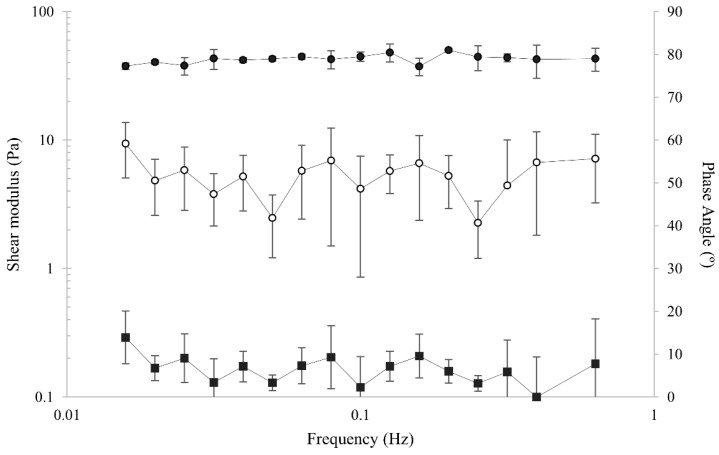
Mechanical spectra of the methacrylated-Kefiran scaffold (2% *w/V*) with shear strain 0.2% at 37 °C. The elastic character of the developed scaffold is confirmed by the observed storage modulus G’ (filled symbol) higher than the loss modulus G’’ (open symbol) and the average 6.4 value obtained from phase angle data (secondary axis).

The MA-Kefiran scaffold rheological behavior was evaluated in this research. From Figure 6 it is possible to observe that the storage modulus is higher than the loss modulus revealing the elastic character of the developed new scaffold.

To evaluate the biomaterial viscoelastic characteristics, the shock absorbing ability is directly correlated to the phase angle (δ) measurements. This estimation is non-correlated to the specimen geometry, since the shift angle, between the response in terms of force and the applied sinusoidal displacement, is being considered [53]. In fact, this phase shift is straightly related to the amount of energy that the scaffold is able to dissipate. In this research, this elastic behavior was quantified by the phase angle, which showed an average value of 6.4. Besides, the storage and loss moduli of the MA-Kefiran scaffold were almost constant between 0.01 Hz and 1 Hz. It is important to point out that besides the *G*′ and *G*″ increase, the average of the phase angle was constant until the end of the experiment. Moreover, the mechanical spectrum obtained (Figure 6), reveals a storage modulus higher than the loss modulus, showing the elastic character of the MA-Kefiran scaffold. Similar data were found with Kefiran scaffolds [16]. Considering the possible TERM applications of MA-Kefiran, the data obtained through the mechanical stimulus derived from the dissipation energy were obtained with only 2% (*w/V*) MA-Kefiran concentrations, revealing that the obtained data can be useful to improve design strategies to create functionalized Kefiran scaffolds with unique properties.

Regarding the adhesion properties of the MA-Kefiran scaffold, it was shown that the newly developed material exhibits robust adhesive behavior comparing to native Kefiran, with values of 0.305 ± 0.067 N/s and 0.135 ± 0.049 N/s, respectively.

It is important to point out that hydrogels are complex hydrophilic polymer networks containing a large amount of water, which results in a complex viscoelastic behavior. These viscoelastic properties are one of the main features to take into consideration in some tissues like bone. In fact, these viscoelastic mechanical properties are important, within the physiological frequency ranges, and particularly over low strain rates [54]. The current research highlighted the fact that the mechanical properties can be perfectly modulated through chemical cross-linking.

### 3.10. Biocompatibility of MA-Kefiran Extracts

Biomaterial-based scaffolds need to be compatible with the cellular components and endogenous cells in host tissues. Hemocompatibility testing of blood-contacting biomaterials is important for a critical evaluation of their in vivo application. In fact, the degree of erythrocyte hemolysis is a key factor in evaluating the biocompatibility of biomaterials [55].

For the hemocompatibility testing, the MA-Kefiran and Kefiran hydrogels were incubated for 4 h with rabbit blood and then the samples were analyzed. According to the American Society for Testing and Materials (ASTM), the materials should not produce a negative effect on immune cells and, in this case, blood cells. According to ASTM, a biomaterial that only bursts 0% to 2% of the blood cells does not have a hemolytic effect. Usually, an appropriate material should not generate more than 2% of hemolysis effect. A higher hemolysis’s degree reveals poor biomaterial’s hemocompatibility [55]. The results demonstrated that the tested Kefiran biopolymer and its modified product (MA-Kefiran) did not present a hemolytic effect (Table 1).

It is important to highlight that Kefiran and MA-Kefiran showed to be suitable biomaterials for medical applications. Nowadays, the hemocompatibility idea is to assure the safety of the biomaterial and to reduce post-implant procedures which ultimately represent a long-term benefit for the patient [56].

**Figure 7:**
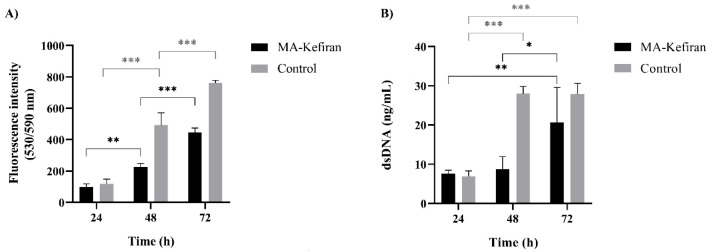
Methacrylated-Kefiran cytotoxicity using L929 cells. (**A**) Metabolic activity of L929 cells in contact with MA-Kefiran extracts during 72 h (AlamarBlue^®^ assay). (**B**) DNA quantification of L929 cells in contact with MA-Kefiran extracts during 72 h. Asterisks denote the significance levels obtained from the Tukey multiple comparison tests applied to the data between time-points: *p* ≤ 0.01 (*), *p* ≤ 0.005 (**), and *p* ≤ 0.0001 (***).

To be a proper biomaterial for in situ tissue regeneration, the chemically modified Kefiran, MA-Kefiran, when in close connection with living tissue should not cause adverse effects. The results of cell viability showed that the MA-Kefiran extract did not present any significant cytotoxic effect on L929 cells at 24 h, 48 h, and 72 h (Figure 7).

Figure 7 revealed that the L929 cells were metabolically active in the presence of the MA-Kefiran and, their ability to grow in the new condition increased during the time of incubation. Therefore, it is important to point out that the degree of methacrylation of the Kefiran polymer did not produce a deleterious effect on cellular metabolic activity; herein, we have demonstrated that Kefiran can be chemically modified and remain biocompatible.

In this research, the results revealed through these validated procedures that this unique MA-Kefiran hydrogel should be evaluated, in the future, in animal models, a step midway between in vitro studies and human clinical trials [57]. The in vivo assessment of the developed hydrogel on OA animal models is currently being performed. The inflammatory response and biocompatibility will first be evaluated by subcutaneous injection of this hydrogel in six weeks old BALB/c mouse. Then, OA will be induced in skeletally mature rabbits though a partial lateral meniscectomy procedure and the therapeutic effect of Kefiran based hydrogels on cartilage degeneration will be evaluated [58].

## 4. Conclusions

The chemical modification can impart specific structure and function to the Kefiran biopolymer, attracting widespread attention for its biomedical applications. Thus, in this research it has been shown that modification of the Kefiran polymer with methacrylic anhydride is feasible and allows to successfully create a new MA-Kefiran hydrogel that was fully characterized. Furthermore, it has been demonstrated that the modified hydrogel was biocompatible and showed an ability to improve cellular function of L929 cells. MA-Kefiran hydrogels, with adjustable rheological properties, and remarkable morphological structure, could represent an interesting candidate for biomedical applications, particularly in TE. Although significant achievements have been made in this research, the application of MA-Kefiran hydrogels in biomedical applications is still emerging and needs further improvement.

## Figures and Tables

**Table 1:** Hemolysis percentage of the methacrylated-Kefiran in contact with blood cells and corresponding American Society for Testing and Materials (ASTM) classification.

Sample	ASTM Classification (%)
MA-Kefiran	1.33 ± 0.21
Kefiran	2.00 ± 0.09
PBS	0

## Data Availability

All data generated/analyzed throughout this research are included in this article.
